# Retraction to “miR-519d downregulates LEP expression to inhibit preeclampsia development”

**DOI:** 10.1515/med-2022-0562

**Published:** 2022-09-19

**Authors:** Hairui Cai, Dongmei Li, Jun Wu, Chunbo Shi

**Affiliations:** Obstetrics and Gynecology Department, Ningbo Women & Children’s Hospital, No. 339, Liuting Road, Ningbo 315000, Zhejiang Province, People’s Republic of China

Retraction of: Cai H, Li D, Wu J, Shi C. miR-519d downregulates LEP expression to inhibit preeclampsia development. Open Med. 2021;16(1):1215–27. DOI: 10.1515/med-2021-0244.

This article has been retracted due to possible irregularities in the data images presented in results discovered by author. His team is working currently on repeat experiment to investigate any other issues of lack of rigor. In order to avoid confusion about scientific issues, all co-authors requested to withdraw the manuscript.

